# The Effects of Qigong and Tai Chi Exercises on Chronic Low Back Pain in Adults: A Systematic Review and Meta-Analysis of Randomized Controlled Trials

**DOI:** 10.7759/cureus.84342

**Published:** 2025-05-18

**Authors:** Spyridon Sotiropoulos, Maria Papandreou, Andreas Mavrogenis, Athanasia Tsaroucha, George Georgoudis

**Affiliations:** 1 Department of Physiotherapy, University of West Attica, Athens, GRC; 2 Musculoskeletal Physiotherapy Research Laboratory, University of West Attica, Athens, GRC; 3 Orthopaedics, School of Medicine, National and Kapodistrian University of Athens, Athens, GRC; 4 1st Department of Anesthesia, Aretaieion University Hospital, National and Kapodistrian University of Athens, Athens, GRC

**Keywords:** chronic low back pain (clbp), pain management, physiotherapy, qigong, systematic review and meta-analysis, taichi

## Abstract

Qigong and Tai Chi are mind-body exercises that may provide therapeutic benefits for individuals with chronic low back pain (CLBP), yet their efficacy remains uncertain. This systematic review and meta-analysis assessed their effects on pain and disability in individuals with CLBP. Randomized controlled trials (RCTs) evaluating Qigong or Tai Chi for CLBP were included. A systematic search was conducted in MEDLINE (Medical Literature Analysis and Retrieval System Online), Embase, Physiotherapy Evidence Database (PEDro), and the Cochrane Library up to September 2023. Pain and disability were the primary outcomes. Risk of bias (RoB) was assessed independently by two reviewers using Cochrane’s RoB 2 tool. The GRADE (Grading of Recommendations Assessment, Development and Evaluation) approach was used to assess the certainty of the evidence. Eight RCTs (n = 729 adults, age range 31.5-73 years, intervention duration 4-24 weeks) were included. Meta-analysis was performed using a random-effects model, with heterogeneity quantified via I² statistics. Eight RCTs (n = 729 participants) were included. Qigong and Tai Chi significantly reduced pain intensity (standardized mean difference (SMD) = -1.07, 95%CI: -1.64 to -0.49, I² = 93%) and disability (SMD = -0.77, 95%CI: -1.39 to -0.15, I² = 93%) compared to control groups. Subgroup analyses suggested greater effect sizes against passive controls (SMD = -1.17, 95%CI: -1.91 to -0.43, I² = 90%) than against active controls (SMD = -0.98, 95%CI: -1.95 to -0.01, I² = 95%). Limitations include substantial heterogeneity among studies and imprecision in effect estimates. The certainty of evidence was rated moderate to high. Qigong and Tai Chi appear effective in reducing pain and disability in individuals with CLBP. However, substantial heterogeneity and the lack of direct comparisons with structured exercise programs necessitate further high-quality RCTs to confirm their long-term effectiveness.

## Introduction and background

Chronic low back pain (CLBP) is a prevalent health concern that significantly impacts individuals globally, being one of the foremost causes of disability [1]. Defined as pain persisting for over three months, CLBP can severely hinder daily activities, occupational performance, and overall quality of life [2]. The lifetime prevalence of low back pain is estimated to be as high as 80%, though only a subset of individuals develop CLBP (defined as pain persisting for more than three months) [3]. This condition not only leads to considerable physical limitations but also results in substantial economic burdens, including healthcare costs and lost productivity due to absenteeism [4,5]. The multifactorial nature of CLBP complicates its pathophysiology, which is often nonspecific and includes various potential causes such as musculoskeletal disorders, degenerative changes, and psychosocial factors [6,7]. Consequently, CLBP reduces quality of life, and its burden extends beyond mere physical discomfort, often leading to comorbidities like sleep disturbances, anxiety, and depression, which further exacerbate the disability experienced by affected individuals [8,9].

In terms of management, conservative treatment approaches are typically prioritized, particularly exercise-based interventions. Exercise therapy has been shown to effectively alleviate pain and enhance function in individuals with CLBP [10]. Various forms of exercise, including physical therapy, strength training, and aerobic conditioning, are recommended as first-line treatments [10,11]. These conservative strategies aim to improve physical function, reduce pain, and promote self-management, thereby minimizing reliance on pharmacological interventions [11]. However, adherence to prescribed exercise regimens can be challenging for many patients, highlighting the need for alternative exercise modalities that may offer additional motivation and engagement [12]. 

Mind-body practices such as Qigong and Tai Chi have gained traction as viable non-pharmacological options for managing CLBP, combining physical movement with mindfulness and relaxation techniques [13]. These traditional Chinese mind-body practices integrate gentle physical movements, breathing exercises, and mental focus, addressing both the physical and psychological dimensions of chronic pain [13,14]. Preliminary studies have indicated that Qigong and Tai Chi may lead to significant reductions in pain intensity and improvements in functional outcomes for individuals suffering from CLBP [13]. Previous reviews have explored traditional Chinese exercises more broadly, but few have specifically focused on randomized controlled trials in CLBP or differentiated Qigong from Tai Chi in relation to control type [13,14]. However, despite the encouraging evidence, systematic evaluations of their efficacy remain limited, necessitating a comprehensive review of the existing literature to better understand their role in managing CLBP and their respective effectiveness against active and passive controls. Moreover, as these practices originate from East Asian cultural contexts, their acceptability and integration into Western healthcare settings may vary, potentially affecting adherence and generalizability of findings.

This systematic review and meta-analysis aims to synthesize the current evidence regarding the effects of Qigong and Tai Chi exercises on CLBP. By evaluating randomized controlled trials (RCTs) that investigate these mind-body practices, this review seeks to clarify their therapeutic potential and provide insights into their effectiveness in pain relief and functional improvement. The findings from this review will contribute to the existing body of literature and may inform clinical practice by highlighting the importance of integrating Qigong and Tai Chi into conventional treatment strategies for individuals with CLBP.

## Review

Methodology

Study Design

This systematic review and meta-analysis adhered to the Preferred Reporting Items for Systematic Reviews and Meta-Analyses (PRISMA) guidelines [15] and followed a pre-registered protocol on the International Prospective Register of Systematic Reviews (PROSPERO) (Registration ID: CRD42022340730). The primary objective was to evaluate the effects of Qigong and Tai Chi interventions on pain intensity and functional disability in CLBP patients.

Search Strategy

A comprehensive literature search was conducted in PubMed/MEDLINE (Medical Literature Analysis and Retrieval System Online), Embase, Cochrane Library, and Physiotherapy Evidence Database (PEDro) databases from inception to September 2023. Search terms included combinations of “chronic low back pain", “Qigong", “Tai Chi", “exercise therapy", and related keywords. The search strategy can be found in Appendix A.

Inclusion Criteria

This review included randomized controlled trials (RCTs) that examined the effects of Qigong and Tai Chi exercises on chronic low back pain. Studies were eligible if they met the following criteria:

Population: Adults aged 18 years or older diagnosed with chronic low back pain, defined as pain persisting for more than three months.

Intervention: Any form of Qigong or Tai Chi exercise delivered through supervised sessions, either in a one-on-one or group format.

Comparison: Studies that included any type of control group, such as active controls (e.g., conventional exercise therapy) or passive controls (e.g., waitlist or no treatment).

Outcome measures: Studies assessing pain intensity and/or functional disability were the focus of this review, but all reported outcomes were considered for data synthesis. All available effect measures for these outcomes were considered in the analysis.

Study Design: Only RCTs were included to ensure high methodological quality, minimize bias, and provide reliable effect estimates.

Language: Only studies published in English were considered.

Exclusion Criteria

Studies were excluded if they met any of the following criteria:

Population: Participants with acute or subacute low back pain (lasting less than three months).

Intervention: Studies in which the active group combined Qigong or Tai Chi with any other treatment modality, such as acupuncture, electrotherapy, manual therapy, or any other physical add-on treatment. Studies where exercises were delivered solely through printed leaflets or videos were also excluded due to the lack of established efficacy compared to therapist-prescribed interventions.

Study Design: Case studies, case series, systematic reviews, and meta-analyses were excluded. However, the citations within systematic reviews and meta-analyses were screened for relevant studies.

Language: Studies published in languages other than English were excluded.

Study Selection

Titles and abstracts of all identified records were independently screened by two reviewers using Rayyan software [16]. Studies deemed potentially relevant were retrieved in full text for further assessment against the eligibility criteria. Disagreements between the reviewers were resolved through discussion. In cases where consensus could not be achieved, a third reviewer acted as an arbitrator to ensure an impartial decision. The inclusion and exclusion criteria were consistently applied during screening and full-text assessment to ensure the inclusion of only eligible studies in the review.

Data Extraction

Following the final selection of studies, two reviewers independently extracted relevant study data using a predesigned Excel spreadsheet to ensure consistency and transparency. The extracted data included trial location, participant characteristics (e.g., age, gender), sample size, inclusion and exclusion criteria, exercise dosage parameters (e.g., frequency, duration, and type of Qigong or Tai Chi exercises), comparator type or control details (e.g., active or passive), and the outcomes assessed. Discrepancies in data extraction between the two reviewers were resolved through discussion, and no need arose for a third reviewer to resolve disagreements.

Risk of Bias Assessment

The risk of bias assessment was conducted using the Cochrane Risk of Bias 2 (RoB 2) tool [17]. This framework evaluates five domains: the randomization process, deviations from intended interventions, missing outcome data, measurement of outcomes, and selection of the reported results. Each domain was assessed independently for all included studies.

Statistical Analysis

A narrative synthesis was initially planned, combined with a meta-analysis. Despite high heterogeneity, meta-analyses were performed to quantitatively summarize the available evidence. Pooled effect sizes for pain intensity and functional disability were calculated. To account for variations in outcome scales across included studies, effect sizes for continuous outcomes were expressed as standardized mean difference (SMD) with 95% confidence intervals (CI) [18]. In accordance with the default approach of Review Manager (RevMan) software (The Cochrane Collaboration, London, United Kingdom), SMD was computed using Hedges' g, which applies a small-sample bias correction to Cohen’s [19]. Given the expected heterogeneity among studies, a random-effects model was employed to pool effect sizes, using the inverse-variance method for weighting individual study estimates [20].

Heterogeneity across studies was assessed using the I² statistic, with thresholds of 25%, 50%, and 75% interpreted as low, moderate, and high heterogeneity, respectively [19]. If high heterogeneity was observed (I² > 75%), subgroup analyses were conducted to explore potential sources of variability, such as intervention type (Qigong vs. Tai Chi) and control type (active vs. passive) [19,20]

Meta-analyses were performed using RevMan software [21]. Subgroup analyses based on intervention type and control group were performed to explore sources of heterogeneity. Forest plots were used to visualize effect sizes and their corresponding CIs, while funnel plots were generated to assess potential publication bias [22]. A Summary of Findings table was produced to provide a transparent overview of the key results. Publication bias was assessed via visual inspection of the funnel plots and using Egger’s regression test and the Trim-and-Fill method with the metafor package in R (R Foundation for Statistical Computing, Vienna, Austria, https://www.R-project.org/).

Certainty of Evidence

The certainty of evidence for each outcome was evaluated using the Grading of Recommendations Assessment, Development, and Evaluation (GRADE) approach. This method assesses the quality of evidence by considering factors such as risk of bias, inconsistency, indirectness, imprecision, and publication bias to determine confidence in effect estimates. Explanations for the GRADE decisions are provided after the Summary of Findings tables to maintain clarity and readability.

Results

**Figure 1:**
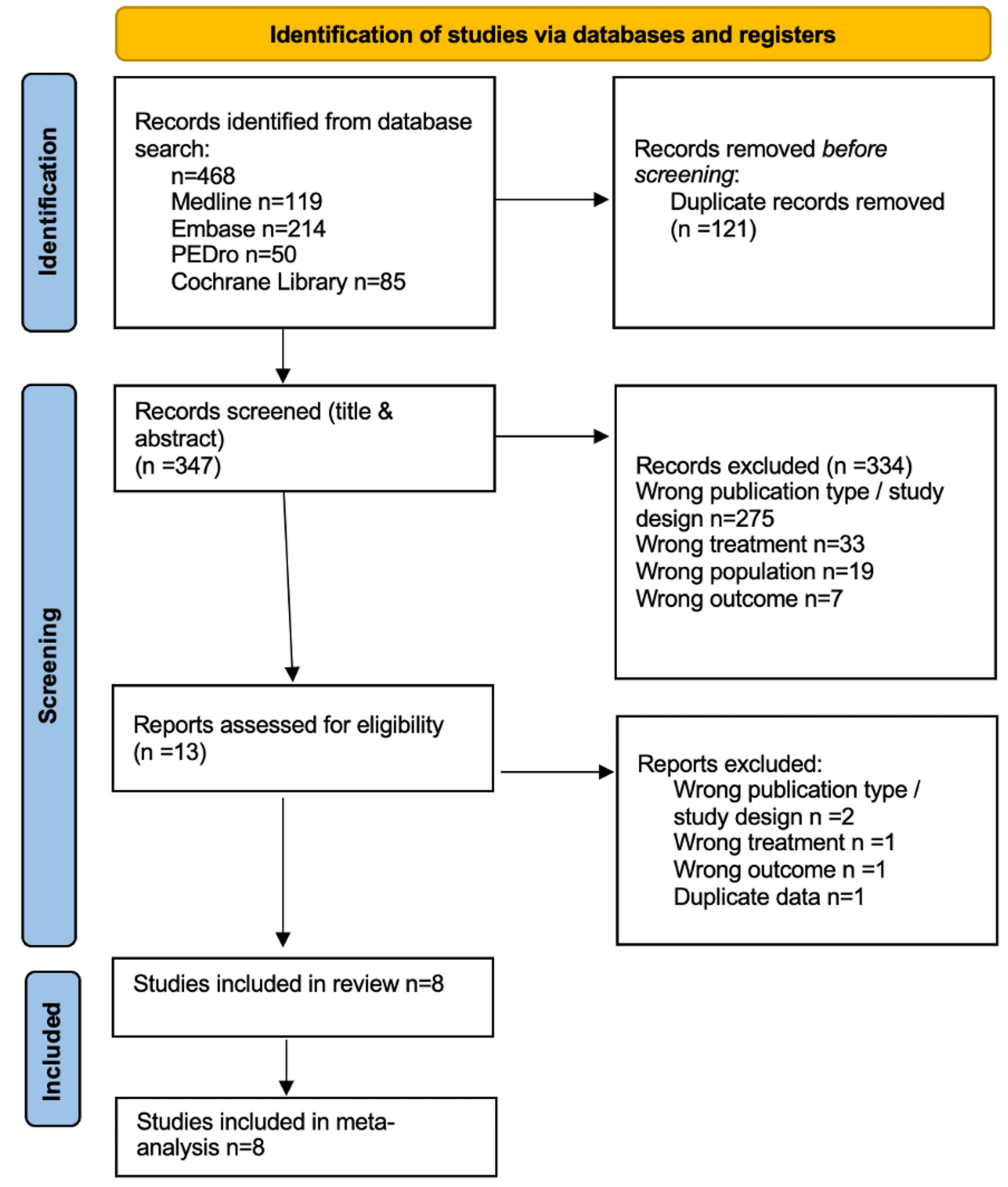
PRISMA flowchart showing study selection PRISMA: Preferred Reporting Items for Systematic Reviews and Meta-Analyses

A total of 468 records were identified through database searches, including MEDLINE (n=119), Embase (n=214), PEDro (n=50), and the Cochrane Library (n=85). After removing 121 duplicate records, 347 studies were screened based on titles and abstracts. Of these, 334 were excluded due to incorrect publication type or study design (n=275), inappropriate treatment comparisons (n=33), irrelevant population (n=19), or non-relevant outcomes (n=7). Thirteen full-text articles were assessed for eligibility, with five additional studies excluded due to study design (n=2), treatment mismatch (n=1), outcome irrelevance (n=1), or duplicate data (n=1). Ultimately, eight studies met the inclusion criteria and were included in both the systematic review and meta-analysis. The PRISMA flow diagram (Figure 1) provides a visual representation of the study selection process [15]. Disagreements between the reviewers were resolved through discussion, and no cases required arbitration by a third reviewer, as consensus was always achieved.

Study Characteristics

Eight RCTs involving 726 participants were included. Interventions ranged from four to 24 weeks, with session durations of 30-90 minutes. Comparators included passive (e.g., waiting list) and active (e.g., core strengthening) controls. Key characteristics are summarized in Table 1.

**Table 1:** Characteristics of the studies included in the review and meta-analysis Exp: Experimental Group, C: Control Group, CCST: Core Stability Training GE: General Exercise, NPRS: Numerical Pain Rating Scale, RMDQ: Roland-Morris Disability Questionnaire, ODI: Oswestry Disability Index, FFbHR: Hannover Functional Ability Questionnaire for measuring back pain-related disability , QBPDS: Quebec Back Pain Disability Scale , PDI: Pain Disability Index ,  PSFS: Patient-Specific Functional Scale, GPE: Global  Perceived Effect, SWSES: Schützler & Witt Self-Efficacy Scale, FRI: Functional Rating Index, ST-5: Srithanya Stress Scale,  PPI: Present Pain Intensity,  PSQI: Pittsburgh Sleep Quality Index , iEMG: Integrated Electromyogram , YBT: Y -Balance Test, EMG: Electromyography

Author (Year)	Sample Size	Age	Experimental	Control	Duration	Measured Outcomes	Adverse Events
Hall et al. (2011) [23]	Exp= 80, C=80, n= 160	44	Tai Chi 2X40 minutes/week	Waitlist	10 weeks	Pain: NPRS; Disability: RMDQ, QQBPDS, PDI, PSFS; Bothersomeness of Back Symptoms: NRS GPE	Minor: 4 participants reported increase in LBP Alleviated before trial ended
Blödt et al. (2015) [24]	Exp= 64, C=63, n= 127	46.7	Qigong 1X90 minutes/week	Strengthening exercises 1X60 minutes/week	3 months	Pain: VAS; Disability: RMDQ; Quality of Life: SF-36; Sleep quality: NRS; Self-efficacy: SWSES	10 patients in both groups Dizziness, increase in pain, muscle soreness, mood fluctuation.
Teut et al. (2016) [25]	Exp= 58, C1=61, C2=57, n= 176	73	Qigong 1X90 minutes/week	C1: Yoga 2X40 minutes/week, C2: Waitlist	3 months	Pain: VAS; FRI Disability: FFbHR; Quality of Life: SF36; Depression: GDS; Strength: Handgrip strength test	Non-Reported
Phattharasupharerk et al. (2019) [26]	Exp= 36, C=36, n= 72	35,3	Qigong 1X60 minutes/week	Waitlist	6 weeks	Pain: VAS; Disability: RMDQ; Back Range of Motion; Core Stability; Index Heart Rate; Respiratory Rate Mental Status: (ST- 5) GPE	Non-Reported
Zou et al. (2019) [27]	Exp= 15, C1=15, C2=13, n= 43	59	Tai Chi 3X60 minutes/week	C1: CST 3X60 minutes/week, C2: Control – no intervention	12 weeks	Pain: VAS' Neuromuscular Functional Assessment: Isokinetic Dynamometer	Non-Reported
Yao et al. (2020) [28]	Exp= 36, C=36, n= 72	53,5	Qigong 4X60 minutes/week	GE 4X60min/week	24 weeks	Pain: VAS, PPI Quality of Life: SF-36 Sleep Quality: PSQI Muscle Strength: iEMG	Non-Reported
Yan et al. (2022) [29]	Exp= 10, C=10, n= 20	69	Tai Chi 3X60 minutes/week	Control group – no intervention	6 weeks	Pain: VAS Gait: Foot scanner Dynamic Balance: YBT	Non-Reported
Yang et al. (2023) [30]	Exp= 29, C=27, n= 56	31.5	Qigong 5X30 minutes/week	Walking 5X30 minutes/week	4 weeks	Pain: NPRS Disability: ODI Surface EMG	Non-Reported

Risk of Bias

The assessment revealed that the overall risk of bias for the included studies ranged from low to moderate. Studies generally demonstrated a low risk of bias in domains related to the randomization process and measurement of outcomes. However, some concerns were identified in domains addressing deviations from intended interventions and missing outcome data, particularly due to the inherent challenges of blinding in exercise-based interventions.

A summary of the risk of bias assessments is provided in Table 2, illustrating the ratings for each domain across all included studies. This structured evaluation ensured a transparent appraisal of the methodological quality of the evidence. (See Appendix B for "Risk of Bias per domain and as percentage (intention to treat)".)

**Table 2:** Risk of bias table D1 = Randomisation process; D2 = Deviations from intended interventions; D3 = Missing outcome data; D4 = Measurement of the outcome; D5 = Selection of the reported result

Author, year	D1	D2	D3	D4	D5	Overall
Phattharasupharek et al., 2018 [26]	Low Risk	Low Risk	Low Risk	Low Risk	Low Risk	Low Risk
Hall et al., 2011 [23]	Low Risk	Low Risk	Low Risk	Some Concerns	Low Risk	Some Concerns
Blödt et al., 2015 [24]	Low Risk	Low Risk	Low Risk	Some Concerns	Low Risk	Some Concerns
Teut et al., 2016 [25]	Low Risk	Low Risk	Low Risk	Some Concerns	Low Risk	Some Concerns
Zou et al., 2019 [27]	Low Risk	Low Risk	Low Risk	Some Concerns	Low Risk	Some Concerns
Yao et al., 2020 [28]	Low Risk	Low Risk	Some Concerns	Some Concerns	Low Risk	Some Concerns
Yan et al., 2022 [29]	Low Risk	Low Risk	Low Risk	Some Concerns	Low Risk	Some Concerns
Yang et al., 2023 [30]	Low Risk	Low Risk	Low Risk	Some Concerns	Low Risk	Some Concerns

Data Synthesis

The included studies varied in study design, intervention duration, and comparator groups. A total of eight RCTs with sample sizes ranging from 20 to 176 participants were analyzed. The interventions primarily included Qigong and Tai Chi, with durations ranging from four to 24 weeks and session frequencies varying from one to five times per week. Control groups differed between active comparators (e.g., conventional physiotherapy, core stabilization exercises) and passive controls (e.g., waiting list or no treatment). Given these variations, a narrative synthesis was conducted to complement the meta-analysis.

Pain Outcomes

All included studies demonstrated that Qigong and Tai Chi exercises produced a statistically significant within-group reduction in pain intensity from baseline; however, not all studies demonstrated statistically significant differences between groups. In comparison with passive controls, Qigong and Tai Chi generally led to greater reductions in pain intensity, supporting their effectiveness in pain management. However, when compared to active controls, such as conventional exercise therapy and core stabilization training, the between-group differences were less pronounced. Some studies found superior pain reduction in Qigong and Tai Chi groups, while others reported no significant differences, suggesting that the effectiveness of these interventions may be comparable to other active treatments rather than clearly superior. The type of comparator played a crucial role in determining the magnitude of the observed effects, with larger effect sizes noted when Qigong and Tai Chi were compared to non-exercise or passive control groups. This variability underscores the need for further research to establish their relative efficacy against structured exercise-based interventions.

Disability

Five studies investigated disability and all demonstrated within-group improvements in disability, but between-group differences varied based on the type of comparator [23-26,30]. Greater reductions in disability were observed when Qigong and Tai Chi were compared to passive controls, while comparisons with active controls, including exercise therapy and core stabilization programs, showed mixed results. Some studies reported significantly greater improvements in disability for Qigong and Tai Chi, while others found no differences between groups, suggesting these interventions may be comparable to structured exercise therapies rather than clearly superior. The variability in findings highlights the need for further research to clarify their relative efficacy in improving functional outcomes.

Quality of Life

Three studies assessed quality of life using the SF-36, all reporting within-group improvements in physical and mental health domains among participants practicing Qigong or Tai Chi. However, between-group differences varied depending on the comparator. Blödt et al. [24] and Teut et al. [25] found significant improvements within the intervention groups, but these gains were comparable to those in the exercise therapy and passive control groups, resulting in no significant between-group differences. In contrast, Yao et al. [28] observed greater improvements in the Qigong group compared to controls, demonstrating a significant between-group effect. These findings suggest that while Qigong and Tai Chi consistently enhance quality of life, their superiority over other active interventions remains inconclusive.

Sleep Quality

Two studies assessed sleep quality, both reporting within-group improvements in participants practicing Qigong or Tai Chi. Yao et al. [28] found significant enhancements in sleep quality as measured by the Pittsburgh Sleep Quality Index (PSQI), with greater improvements in the intervention group compared to controls. Blödt et al. [24] also observed improvements, though between-group differences were not statistically significant, suggesting that while both may enhance sleep, their advantage over other interventions remains uncertain.

Neuromuscular Function

Three studies assessed neuromuscular function in individuals with chronic low back pain. Zou et al. [27] found that Tai Chi improved lower-limb neuromuscular endurance compared to a control group, with similar effects observed in core stability training. Yao et al. [28] reported significant within-group improvements in trunk muscle activity in the Wuqinxi group, with greater gains compared to a general exercise control. Yang et al. [30] found that Baduanjin exercise enhanced lumbar erector spinae function, with superior improvements in electromyographic parameters compared to a walking exercise group. While all interventions led to neuromuscular improvements, between-group differences varied, suggesting that Qigong and Tai Chi may provide comparable benefits to other active rehabilitation exercises.

Psychological Well-being

Only two studies examined psychological well-being. Teut et al. [25] found significant within-group reductions in depression (GDS) with Qigong but no between-group differences when compared with yoga exercises. Phattharasupharerk et al. [26] reported similar within-group stress reductions (ST-5) without significant differences from controls. While Qigong and Tai Chi may reduce distress, their advantage over other interventions remains uncertain.

Adverse Events

Three studies reported adverse events related to Qigong and Tai Chi interventions. Blödt et al. [24] and Teut et al. [25] found no serious adverse events, with only minor and transient discomfort reported by some participants. Yao et al. [28] similarly reported no significant safety concerns. These findings support the safety of Qigong and Tai Chi as a treatment option for individuals with chronic low back pain.

Meta-Analysis

**Figure 2:**
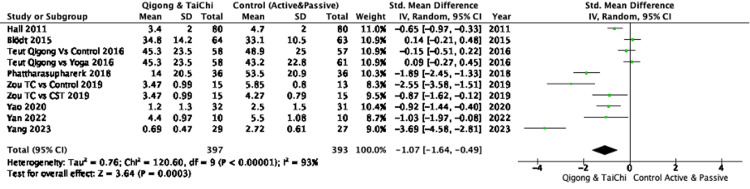
Forest plot of SMD in pain intensity between Qigong/Tai Chi and control groups (active or passive) Negative values indicate a reduction in pain favoring the intervention. Pain outcomes were measured using scales such as NPRS, VAS, and PPI SMD: Standardized Mean Difference, NPRS: Numeric Pain Rating Scale, VAS: Visual Analog Scale, PPI: Present Pain Intensity References: [23-30]

**Figure 3:**
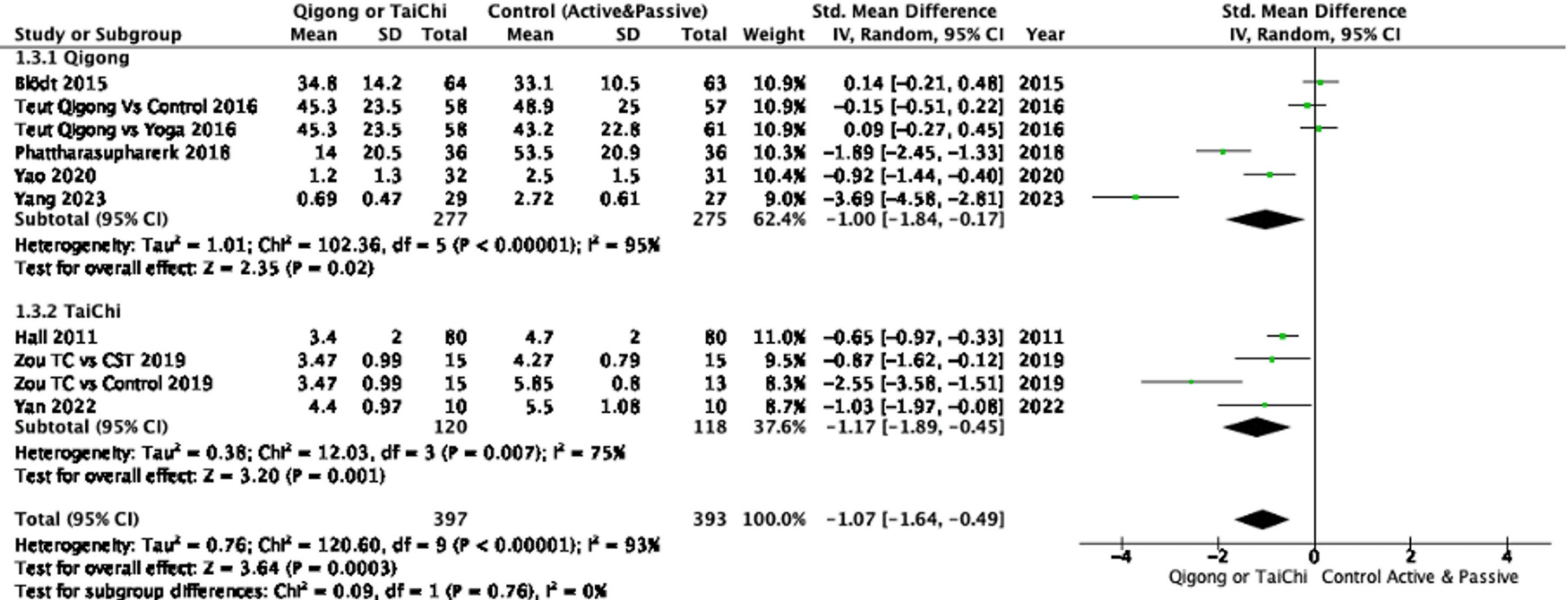
Forest plot of SMDs in pain intensity comparing Qigong and Tai Chi interventions with active or passive controls Results are presented separately for Qigong and Tai Chi. Negative values indicate a reduction in pain favoring the intervention. Pain intensity was measured using scales such as the VAS, NPRS, and PPI. SMD: Standardized Mean Difference, NPRS: Numeric Pain Rating Scale, VAS: Visual Analog Scale, PPI: Present Pain Intensity References:  [23-30]

**Figure 4:**
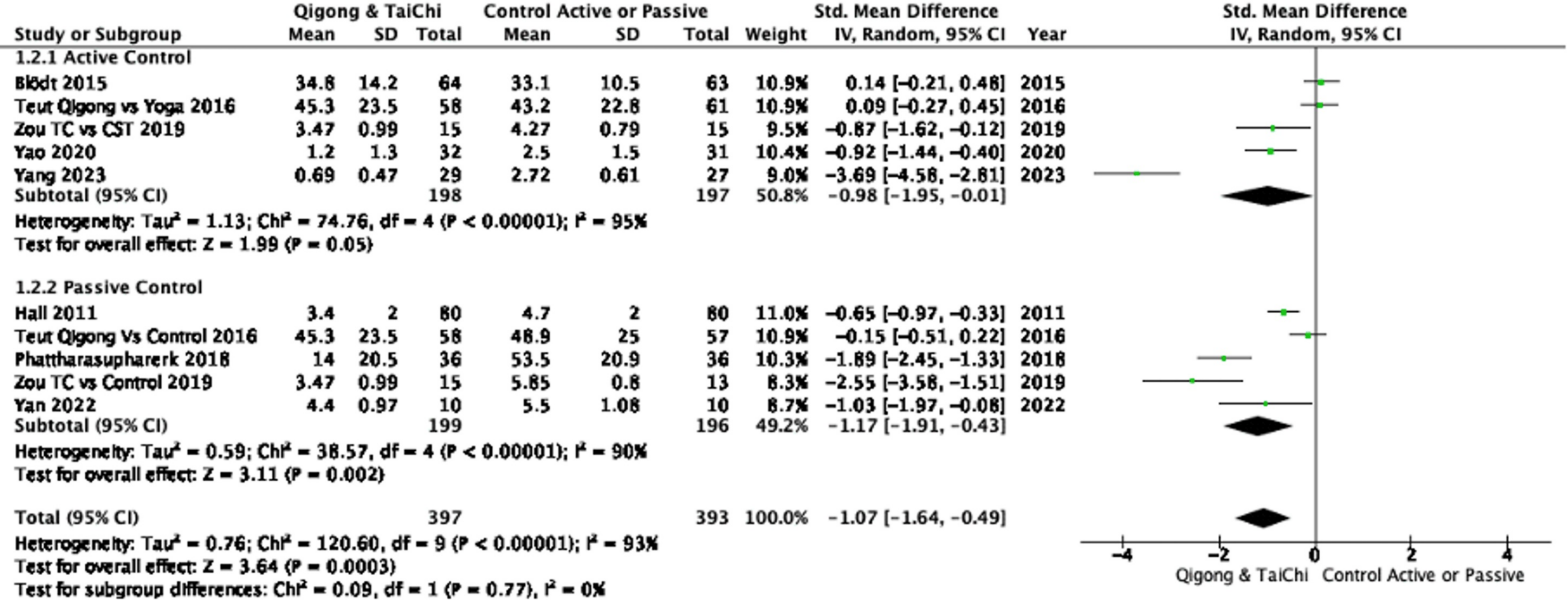
Forest plot of SMDs in pain intensity comparing Qigong and Tai Chi interventions with either active controls (e.g., exercise therapy) or passive controls (e.g., waitlist or no treatment). Negative values indicate greater reductions in pain favoring the intervention. Pain intensity was measured using scales such as the VAS, NPRS, and PPI. A random-effects model was used. Heterogeneity was substantial in both subgroups (I² = 95% for active controls; I² = 90% for passive controls). The test for subgroup differences was not statistically significant (p = 0.77). SMD: Standardized Mean Difference, NPRS: Numeric Pain Rating Scale, VAS: Visual Analog Scale, PPI: Present Pain Intensity References: [23-30]

**Figure 5:**
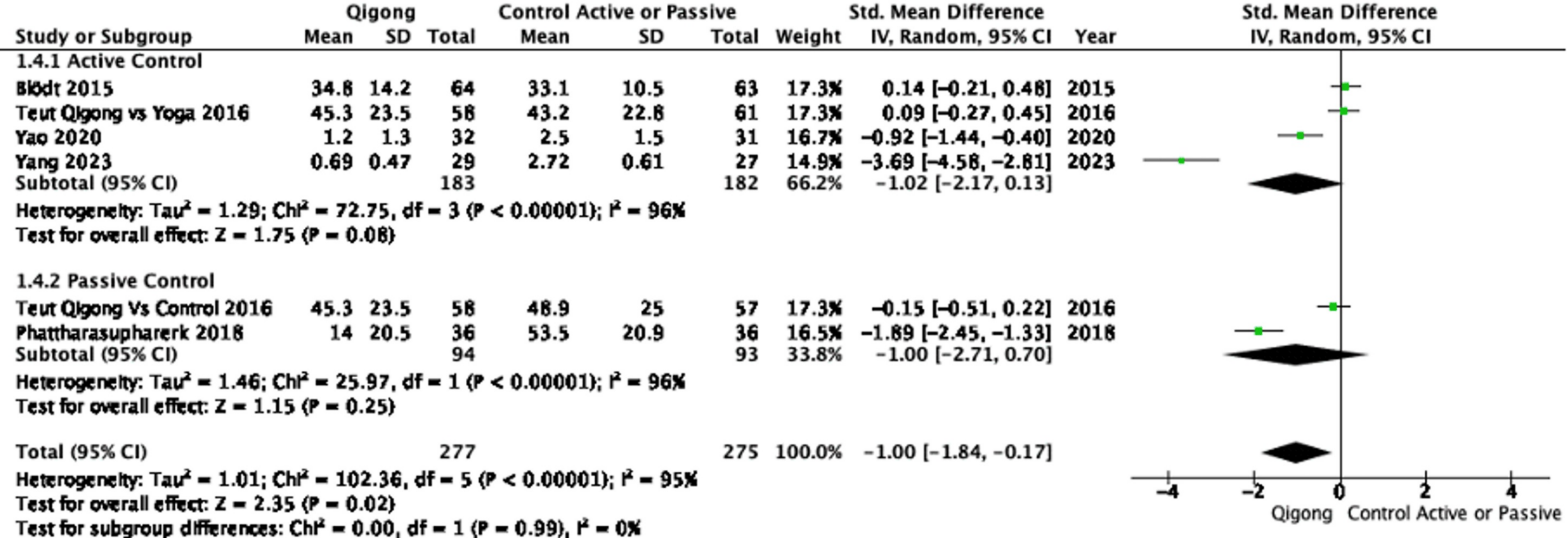
Forest plot of SMDs in pain intensity comparing Qigong interventions with either active controls (e.g., yoga, exercise) or passive controls (e.g., waitlist or no treatment). Negative values indicate a reduction in pain favoring Qigong. Pain intensity was assessed using validated instruments such as the VAS, NPRS, and PPI. A random-effects model was used. SMD: Standardized Mean Difference, NPRS: Numeric Pain Rating Scale, VAS: Visual Analog Scale, PPI: Present Pain Intensity References: [24-26,28,30]

**Figure 6:**
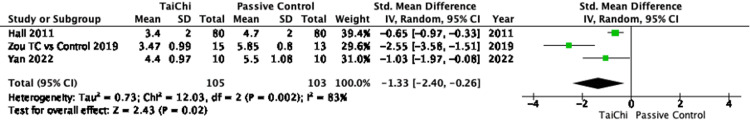
Forest plot of SMDs in pain intensity comparing Tai Chi interventions with passive controls (e.g., waitlist or no treatment) Negative values indicate greater reductions in pain favoring Tai Chi. Pain intensity was measured using validated scales such as the VAS and NPRS. A random-effects model was used. SMD: Standardized Mean Difference, NPRS: Numeric Pain Rating Scale, VAS: Visual Analog Scale References: [23,27,29]

Pain relief: The pooled analysis of eight RCTs demonstrated that participants practicing Qigong or Tai Chi experienced a significant reduction in pain intensity compared to controls, with an SMD of -1.07 (95%CI: -1.64 to -0.49), indicating a large effect. However, substantial heterogeneity was observed (I² = 93%), suggesting variability across studies (Figure 2). Subgroup analyses showed that Qigong, compared to both active and passive controls, resulted in significant pain reduction (SMD: -1.00; 95%CI: -1.84 to -0.17), with high heterogeneity (I² = 95%). Tai Chi demonstrated a substantial pain reduction compared to controls (SMD: -1.17; 95%CI: -1.89 to -0.45) with lower, though still substantial, heterogeneity (I² = 75%) (Figure 3). When comparing Qigong and Tai Chi to active controls, the pooled effect size was large (SMD: -0.98, 95%CI: -1.95 to -0.01), but the CI approached the null effect, indicating some imprecision. Against passive controls, the effect was greater (SMD: -1.17; 95% CI: -1.91 to -0.43), though high heterogeneity (I² = 90%) was also present (Figure 4). Further subgroup analyses revealed that Qigong, compared to active controls, resulted in a large effect size (SMD: -1.02; 95%, CI: -2.17 to 0.13), but the confidence interval crossed the null effect, suggesting imprecision, with very high heterogeneity (I² = 96%). Against passive controls, Qigong demonstrated a large effect (SMD: -1.00; 95%CI: -2.71 to -0.70), though heterogeneity remained high (I² = 96%) (Figure 5). Tai Chi, compared to passive controls, also showed a significant reduction in pain (SMD: -1.33; 95%CI: -2.40 to -0.26), but small sample sizes and wide confidence intervals contributed to imprecision (Figure 6).

**Figure 7:**
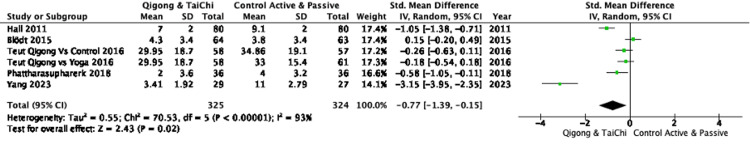
Forest plot of SMDs in disability comparing Qigong and Tai Chi interventions with active or passive controls Negative values indicate reduced disability favoring the intervention. Disability was measured using instruments such as the RMDQ, ODI, and FFbHR. SMD: Standardized Mean Difference, RMDQ: Roland-Morris Disability Questionnaire, ODI: Oswestry Disability Index, FFbHR: Hannover Functional Ability Questionnaire References: [23-26,30]

**Figure 8:**
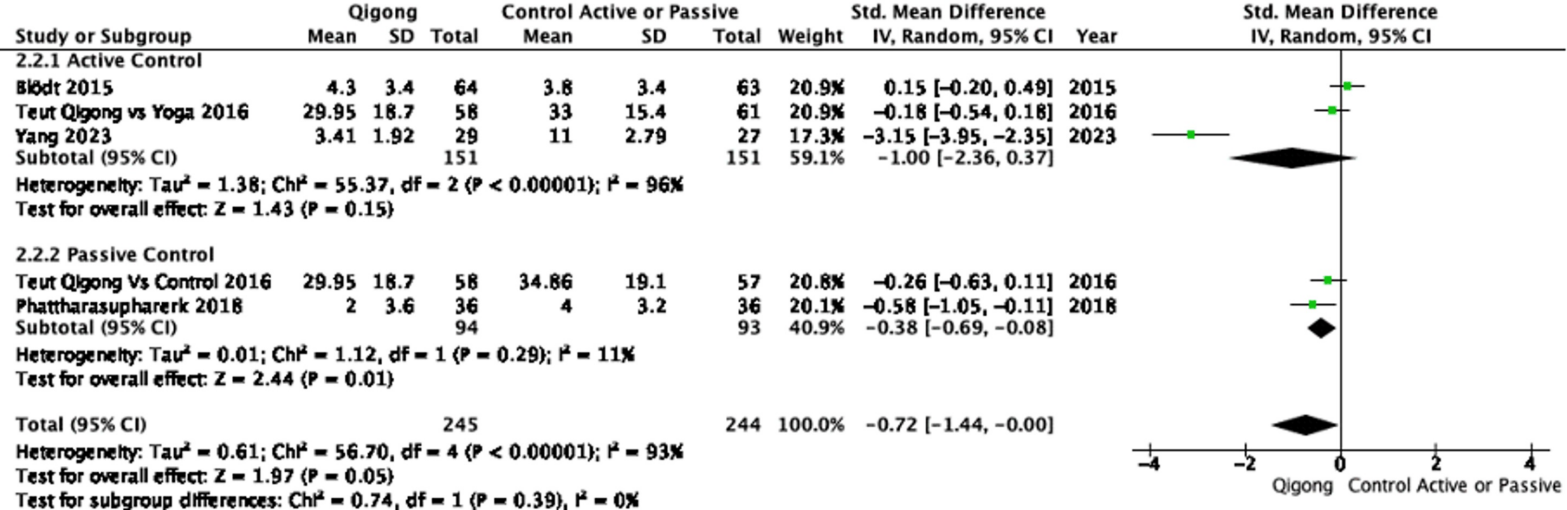
Forest plot of SMDs in disability comparing Qigong interventions with active controls (e.g., yoga) and passive controls (e.g., waitlist or no treatment) Negative values indicate reduced disability favoring Qigong. Disability was assessed using validated instruments such as the RMDQ, ODI, and FFbHR. A random-effects model was used. RMDQ: Roland-Morris Disability Questionnaire, ODI: Oswestry Disability Index, FFbHR: Hannover Functional Ability Questionnaire References: [24-26,30]

Disability:In order to ensure consistency across disability measures, Hannover Functional Ability Questionnaire for measuring back pain-related disability (FFbHR) scores were inverted (100 - original score) so that higher values represented greater disability, aligning with Roland-Morris Disability Questionnaire (RMDQ) and Oswestry Disability Index (ODI), following Cochrane Handbook guidelines [22]. Five RCTs reported moderate improvements following Qigong and Tai Chi compared to controls, with a pooled effect size of -0.77 (95% CI: -1.39 to -0.15) (Figure 7) [23-26,30]. However, heterogeneity remained high (I² = 93%), likely due to differences in intervention protocols and disability measurement tools. When evaluating Qigong alone compared to both active and passive controls, the effect size was -0.72 (95%CI: -1.44 to 0.00), with the confidence interval including 0, indicating imprecision. Against active controls, Qigong resulted in a large improvement (SMD: -1.00; 95%CI: -2.36 to -0.37), but high heterogeneity was observed (I² = 95%). In contrast, when compared to passive controls, Qigong showed a smaller improvement (SMD: -0.38; 95%CI: -0.69 to -0.08) (Figure 8).

To further assess the risk of publication bias, we conducted Egger’s regression test and the Trim-and-Fill method using the metafor package in R [31]. Egger’s test detected potential funnel plot asymmetry (t = -3.3159, p = 0.0106) [32], suggesting possible small-study effects. However, since Egger’s test is less reliable for meta-analyses with fewer than 10 studies [33], we interpreted this result with caution. Therefore, we used the Trim-and-Fill method, which estimated zero missing studies, indicating that the asymmetry detected by Egger’s test may not significantly impact the pooled effect size [34]. Given the small number of included studies, high heterogeneity, and the absence of missing studies in Trim-and-Fill analysis, we determined that publication bias was not a major concern (see Discussion). Therefore, we did not downgrade the certainty of evidence for publication bias in the GRADE [35].

Summary of Findings

The GRADE assessment rated evidence for pain relief as high certainty and for disability improvement as moderate certainty due to heterogeneity. Although blinding of participants and therapists was inherently challenging in exercise interventions, this was not deemed serious enough to downgrade for risk of bias. However, substantial heterogeneity led to downgrades for inconsistency in several comparisons. Some analyses were also downgraded for imprecision due to small sample sizes and wide confidence intervals. Funnel plots were assessed for potential publication bias and are available in Appendix C. The Summary of Findings table for pain relief and disability (Table 3 and Table 4, respectively) provides a detailed overview of the main outcomes, effect sizes, and certainty ratings.

**Table 3:** Summary of findings – Pain Relief NOTE: a. Blinding was not feasible for exercise interventions; high performance bias was therefore not considered. Detection bias did not result in significant outcome differences. b. Substantial heterogeneity (I² = 93%); subgroup analyses (active: I² = 95%, passive: I² = 90%) did not explain variability. Differences in control types and effect size variations likely contributed. No significant subgroup difference (p = 0.77). c. Downgraded for serious inconsistency (I² = 95%) and varied effect sizes; effects ranged from small to very large, though consistently favoring Qigong. d. Funnel plot showed asymmetry, suggesting possible publication bias. e. High heterogeneity and effect size variability due to differing control types. f. Substantial heterogeneity and wide effect size variation. g. Borderline significance; sample size slightly below 400 with wide CI. h. High heterogeneity, non-overlapping CIs, and asymmetric funnel plot. i. Total sample slightly below 400; groups each under 200. j. Funnel plot asymmetry suggests potential missing small studies. k. Heterogeneity (I² = 96%) and non-overlapping CIs indicating outcome variability. l. CI crosses null effect; estimate uncertain with wide CI, high heterogeneity, and slightly small sample size. m. Very high heterogeneity (I² = 96%), non-overlapping CIs, and substantial effect size variation. n. Small sample and wide CI crossing the null effect line. o. Substantial heterogeneity (I² = 82%) and effect size variation. p. Small sample (N = 208) and wide CI, limiting certainty about effect magnitude. CI: confidence interval; SMD: standardised mean difference

Certainty assessment							№ of patients		Effect		Certainty	Importance
№ of studies	Study design	Risk of bias	Inconsistency	Indirectness	Imprecision	Other considerations	Qigong and Taichi	Active and Passive Controls	Relative (95% CI)	Absolute (95% CI)		
Pain relief -Main outcome												
8	randomised trials	not serious^a^	serious^b^	not serious	not serious	strong association	397	393	-	SMD 1.07 SD lower (1.64 lower to 0.49 lower)	⨁⨁⨁⨁ High^a,b^	
Subgroup: Qigong vs Control (Active & Passive)												
5	randomised trials	not serious	serious^c^	not serious	not serious	publication bias strongly suspected strong association^d^	277	275	-	SMD 1 SD lower (1.84 lower to 0.17 lower)	⨁⨁⨁◯ Moderate^c,d^	
Subgroup: Tai Chi vs Control (Active & Passive)												
3	randomised trials	not serious	serious^e^	not serious	not serious	strong association	120	118	-	SMD 1.17 SD lower (1.89 lower to 0.45 lower)	⨁⨁⨁⨁ High^e^	
Subgroup: Combined Qigong & Tai Chi vs Active Control												
5	randomised trials	not serious	serious^f^	not serious	serious^g^	strong association	198	197	-	SMD 0.98 SD lower (1.95 lower to 0.01 lower)	⨁⨁⨁◯ Moderate^f,g^	
Subgroup: Combined Qigong & Tai Chi vs Passive Control												
5	randomised trials	not serious	serious^h^	not serious	not serious^i^	strong association^j^	199	196	-	SMD 1.17 SD lower (1.91 lower to 0.43 lower)	⨁⨁⨁⨁ High^h,i,j^	
Subgroup: Qigong vs Active Control												
4	randomised trials	not serious	serious^k^	not serious	serious^l^	strong association	183	182	-	SMD 1.02 SD lower (2.17 lower to 0.13 higher)	⨁⨁⨁◯ Moderate^k,l^	
Subgroup: Qigong vs Passive Control												
2	randomised trials	not serious	serious^m^	not serious	serious^n^	strong association	94	93	-	SMD 1 SD lower (2.71 lower to 0.7 higher)	⨁⨁⨁◯ Moderate^m,n^	
Subgroup: Tai Chi vs Passive Control												
2	randomised trials	not serious	serious^o^	not serious	serious^p^	strong association	105	103	-	SMD 1.33 SD lower (2.4 lower to 0.26 lower)	⨁⨁⨁◯ Moderate^o,p^	

**Table 4:** Summary of findings – Disability NOTE: a. Concerns regarding detection bias from the lack of blinded outcome assessors, which could influence subjective measures such as disability scores. Performance bias was not considered due to the inherent challenges of blinding in exercise-based interventions. b. High heterogeneity, wide range in effect sizes. c. Substantial heterogeneity and a wide range of effect sizes. d. Confidence interval includes both clinically relevant benefits and no effect, indicating uncertainty about the precision of the estimated effect. e. Substantial heterogeneity and poor overlap of confidence intervals across studies. Variability in the magnitude and direction of the effect. f. 95% CI crosses the line of no effect (SMD = 0). Total sample size is below the recommended threshold for continuous outcomes. g. Lack of assessor blinding in one of the two studies, increasing the risk of detection bias. h. Wide confidence interval close to no-effect line and small sample size, indicating uncertainty in the effect estimate. CI: confidence interval; SMD: standardized mean difference

Certainty assessment							№ of patients		Effect		Certainty	Importance
№ of studies	Study design	Risk of bias	Inconsistency	Indirectness	Imprecision	Other considerations	Qigong and Taichi	Active and Passive Controls	Relative (95% CI)	Absolute (95% CI)		
Disability Improvement												
5	randomised trials	not serious^a^	serious^b^	not serious	not serious	strong association	325	324	-	SMD 0.77 SD lower (1.39 lower to 0.15 lower)	⨁⨁⨁⨁ High^a,b^	
Disability Subgroup: Qigong vs Control (active & Passive)												
4	randomised trials	not serious	serious^c^	not serious	serious^d^	none	151	151	-	SMD 0.72 SD lower (1.44 lower to 0 )	⨁⨁◯◯ Low^c,d^	
Subgroup Disability Qigong vs Active Control												
3	randomised trials	not serious	serious^e^	not serious	serious^f^	strong association	151	151	-	SMD 1 SD lower (2.36 lower to 0.37 higher)	⨁⨁⨁◯ Moderate^e,f^	
Subgroup Disability: Qigong vs Passive Control												
2	randomised trials	serious^g^	not serious	not serious	serious^h^	none	94	93	-	SMD 0.38 SD lower (0.69 lower to 0.08 lower)	⨁⨁◯◯ Low^g,h^	

Discussion

Summary of Key Findings

This systematic review and meta-analysis examined the effectiveness of Qigong and Tai Chi in managing CLBP. The findings suggest that both interventions significantly reduce pain and disability compared to control conditions, providing moderate to high-quality evidence supporting their efficacy, though the certainty of evidence varied across outcomes. Subgroup analyses suggested that differences in intervention type (Qigong vs. Tai Chi) and control group type (active vs. passive) contributed to heterogeneity. While Qigong and Tai Chi both demonstrated significant pain reduction, the effect sizes varied depending on the comparator, with higher effects seen against passive controls compared to active controls. The high heterogeneity observed in these comparisons highlights the need for more standardized research designs to improve comparability across studies.

Pain Outcomes

Our findings align with existing systematic reviews and meta-analyses evaluating Qigong and Tai Chi for musculoskeletal pain. Li et al. conducted a network meta-analysis comparing 20 different exercise interventions, which included both RCTs and quasi-experimental studies, finding that Tai Chi (SMD = -2.11, 95%CI: −3.62 to −0.61) was among the most effective interventions for CLBP relief compared to conventional rehabilitation [36]. However, the inclusion of quasi-experimental studies in their analysis may affect internal validity. Furthermore, our review provided, through subgroup analysis, a clearer distinction between Qigong and Tai Chi’s effects across different control conditions

Similarly, Kong et al. analyzed a mix of small-scale RCTs and observational studies, identifying a significant pain reduction effect of Tai Chi for CLBP (SMD = -0.81; 95%CI: -1.11 to -0.52) [37]. However, their review included heterogeneous chronic pain conditions, making it difficult to isolate Tai Chi’s specific effects on CLBP, as only three studies were investigating CLBP patients. Additionally, their analysis relied on trials with varying intervention durations and control comparisons, contributing to inconsistencies in the reported effects. Our review addresses these limitations by focusing solely on CLBP, applying stricter inclusion criteria for methodological quality, and differentiating Qigong and Tai Chi across passive and active controls, allowing for a more precise evaluation of their therapeutic benefits. Hall et al. conducted a systematic review and meta-analysis that provided moderate-quality evidence supporting Tai Chi for chronic musculoskeletal pain [38]. They reported significant short-term reductions in pain (SMD = -0.66, 95% CI: -0.85 to -0.48), findings that closely align with our results, even though our study only focused on CLBP.

Zhang et al. conducted a meta-analysis that included studies with varying control conditions, reporting that traditional Chinese exercises, including Qigong and Tai Chi, significantly improved pain (SMD = -0.64, 95%CI: −0.90 to −0.37) [39]. Similar to our meta-analysis, they demonstrated a greater effect when compared to passive interventions than when compared to active interventions, supporting the notion that while these practices are beneficial, their superiority over other active treatments remains less conclusive. However, their review did not distinguish the differences between Qigong and Tai Chi, as well as the differences arising from controls.

Disability Outcomes

The beneficial effects of Qigong and Tai Chi extend beyond pain reduction to improvements in disability. Zhang et al. found that traditional Chinese exercises significantly improved disability scores (ODI = -0.96, 95%CI: −1.42 to −0.50) [39]. Hall et al. identified a significant effect of Tai Chi on disability reduction (SMD = -0.66, 95% CI: -0.85 to -0.46), which is comparable to our results [38]. Our meta-analysis demonstrated a pooled SMD for Qigong compared to active or passive controls of -0.72 (95%CI: -1.44, -0.00, I² = 93%), while the combined effect for Qigong and Tai Chi was slightly larger (SMD = -0.77, 95%CI: -1.39, -0.15, I² = 93%). Notably, they reported moderate heterogeneity (I² ≥ 50%), whereas our study observed higher heterogeneity (I² = 93%), particularly in subgroup analyses for Qigong and Tai Chi vs. active controls (I² = 95%). This could be explained by the diversity of Qigong and Tai Chi programs implemented and the diversity of the comparators. Our review enhances understanding of Qigong and Tai Chi for disability reduction by incorporating subgroup analyses that explore the impact of intervention type and comparator groups. This approach provides a more detailed evaluation of their effectiveness in different clinical contexts.

Potential Mechanisms of Action

The beneficial effects of Qigong and Tai Chi on CLBP are multifaceted, involving biomechanical, neurophysiological, and psychological components. These practices are believed to modulate pain perception through the activation of descending pain inhibitory pathways, leading to increased endogenous opioid release and modulation of pain signals via the periaqueductal gray matter (PAG) and the rostral ventromedial medulla (RVM) [40,41]. Studies suggest that Qigong and Tai Chi stimulate these pathways, enhancing pain inhibition and reducing central sensitization [42]. Additionally, these interventions may enhance cortical plasticity, particularly within sensorimotor regions, leading to improved proprioception and altered pain modulation [43]. Tai Chi incorporates slow, controlled movements, which enhance postural control, core stability, and lower limb strength, contributing to spinal stabilization and pain relief [14]. Qigong integrates breath control and mindfulness, which have been shown to downregulate the hypothalamic-pituitary-adrenal (HPA) axis, reducing stress-related hyperalgesia [44]. This modulation of stress-induced pain sensitivity through breathwork is a key component of Qigong's therapeutic effects. Qigong and Tai Chi have also been linked to increased vagal tone, which enhances autonomic regulation and reduces inflammation, both of which are critical factors in chronic pain management [45,46].

These mind-body exercises may also reduce pain-related fear-avoidant behaviors and improve psychological well-being, essential components of a comprehensive CLBP management approach. Studies suggest that Qigong and Tai Chi may enhance neuromuscular function, improving lower-limb strength and reducing pain-related movement impairments [47]. Similarly, Yang et al. demonstrated that Baduanjin Qigong improved lumbar muscle function, suggesting a stabilization effect on the spine, which could further explain reductions in CLBP [30]. The combination of neurophysiological, biomechanical, and psychological adaptations provides a plausible explanation for the pain relief and functional improvements observed in CLBP patients practicing Qigong and Tai Chi. Future research should continue to investigate these mechanisms using neuroimaging, electromyography, and inflammatory biomarker analyses to further validate these effects.

Methodological Limitations

Despite promising results, several methodological limitations must be acknowledged. Substantial heterogeneity (I² = 93%) was observed, likely due to variability in study populations, intervention durations (ranging from four to 24 weeks), exercise styles (Qigong vs. Tai Chi), and control group types (active vs. passive). Subgroup analyses confirmed that heterogeneity remained high (I² = 95% for Qigong vs. active controls; I² = 90% for comparisons with passive controls), suggesting that control group selection does not fully account for this variability. Additional unexplored factors, such as session frequency and adherence rates, may further contribute to heterogeneity. Second, many included studies had small sample sizes (ranging from 20 to 176 participants per study), limiting statistical power and increasing the risk of effect size overestimation. Furthermore it was chosen not to perform a sensitivity analysis due to the small number (n=8) of included studies, as excluding individual studies could have significantly altered the pooled estimate, leading to unstable and unreliable results, as small sample sizes in the absence of sufficient validation data, can amplify statistical uncertainty [48].

While Qigong and Tai Chi demonstrated significant benefits, their superiority over other active interventions remains uncertain. Against passive controls, effect sizes were larger (SMD = -1.17, 95%CI: -1.91 to -0.43), while comparisons with active controls yielded more variable results (SMD = -0.98, 95% CI: -1.95 to -0.01), with wider confidence intervals suggesting uncertainty. Few studies directly compared different styles or intensities of Qigong and Tai Chi, making it difficult to establish optimal intervention parameters. Subgroup analyses (Qigong vs. Tai Chi) suggested some differences in functional outcomes, with Tai Chi favoring disability improvement (SMD = -0.66) and Qigong favoring pain reduction (SMD = -1.00). However, the absence of head-to-head trials limits the reliability of these findings, underscoring the need for direct comparative studies.

Publication bias cannot be excluded. Visual inspection of funnel plots (Appendix C) and Egger’s test suggested the possibility of publication bias. However, given the limited number of studies, the reliability of this assessment remains uncertain [33]. It was decided that the GRADE approach would not formally apply a downgrade for publication bias, as the Trim-and-Fill method detected no missing studies [34]. The possibility of small study effects cannot be conclusively ruled out, as smaller trials with large effect sizes may have been preferentially published. Therefore, future studies with prospective trial registration and larger sample sizes could be used to better assess and minimize potential publication bias.

This review excluded observational studies, quasi-experimental designs, and case series, which may offer insights into long-term adherence and real-world effectiveness. However, these study types are more prone to bias and confounding, so future research should complement RCT evidence with broader study designs to enhance external validity. Only articles published in English were included, and we are aware that this may introduce language bias and exclude relevant studies from non-English journals.

Clinical and Research Implications

The findings of this meta-analysis support the inclusion of Qigong and Tai Chi as non-pharmacological treatment options for individuals with CLBP, similar to other active exercise treatments. However, given the methodological limitations and high heterogeneity across studies, clinicians should be cautious in applying these findings to practice before further data are published.

## Conclusions

This systematic review and meta-analysis provide moderate-quality evidence supporting the efficacy of Qigong and Tai Chi in reducing pain and disability in individuals with CLBP. However, the substantial heterogeneity, small sample sizes, and self-reported outcomes suggest caution in the interpretation of pooled effects. Publication bias and imprecision remain possible, and generalizability is constrained by language restrictions and the lack of diverse populations. While studies comparing these interventions to passive controls demonstrated significant benefits, the superiority over active controls remains less conclusive. Further high-quality RCTs with standardized protocols, well-defined control groups, head-to-head comparisons, and long-term follow-ups are needed to refine clinical recommendations and determine sustained effectiveness and safety.

## Figures and Tables

**Figure 9:**
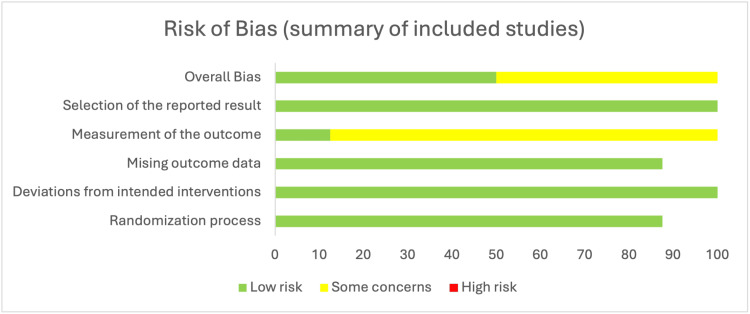
Risk of Bias per domain

**Figure 10:**
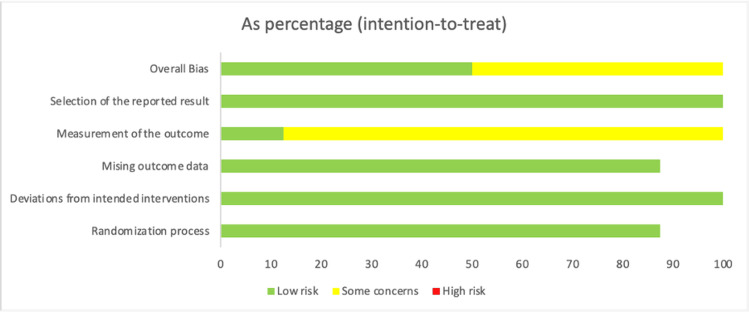
Risk of Bias as percentage

**Figure 11:**
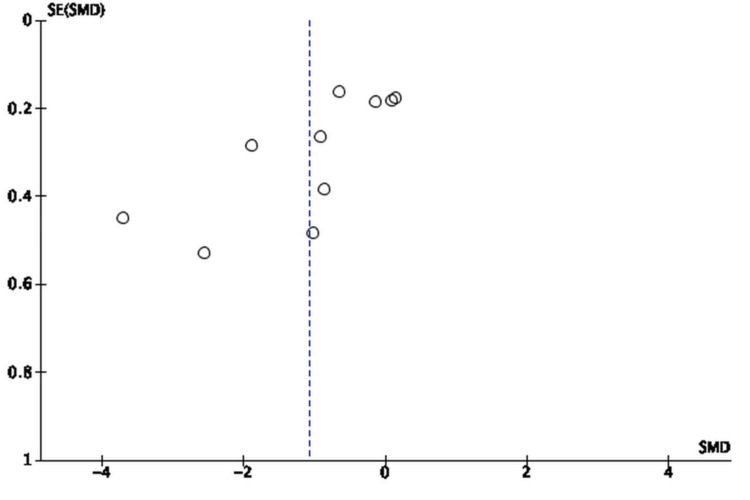
Funnel plot for combined Qigong and TaiChi exercises vs active and passive controls References: [23–30]

**Figure 12:**
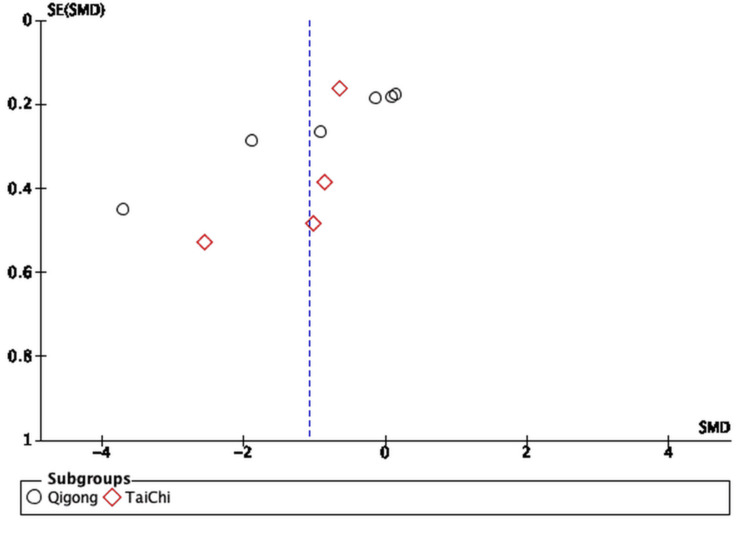
Funnel plot for subgroups Qigong or Tai Chi vs active and passive controls References: [23–30]

**Figure 13:**
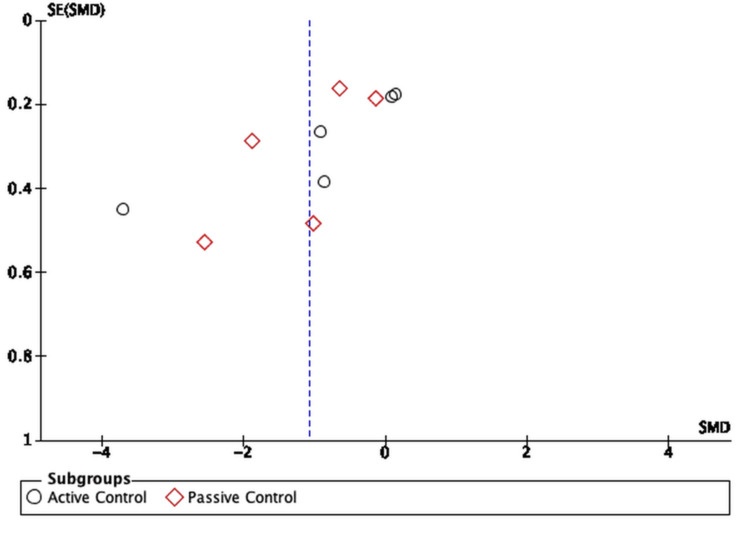
Funnel Plot for combined Qigong and Tai Chi exercises vs active or passive controls References: [24–26,28,30]

**Figure 14:**
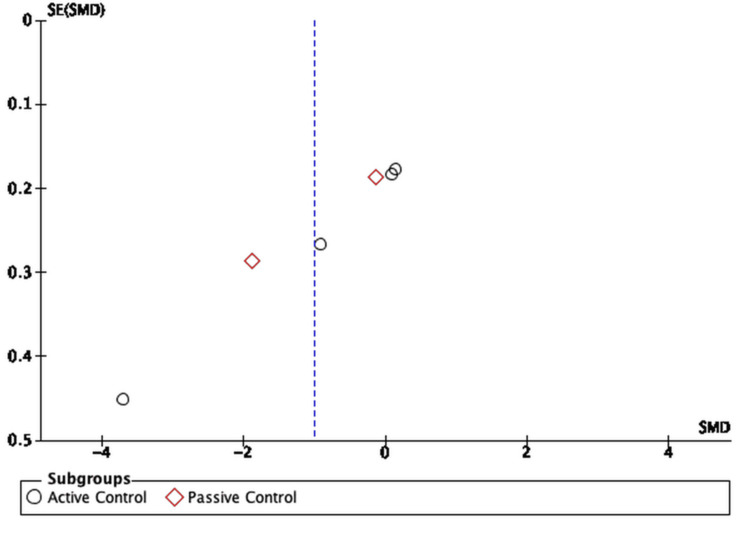
Funnel plot Qigong vs active or passive controls References: [23,27,29]

**Figure 15:**
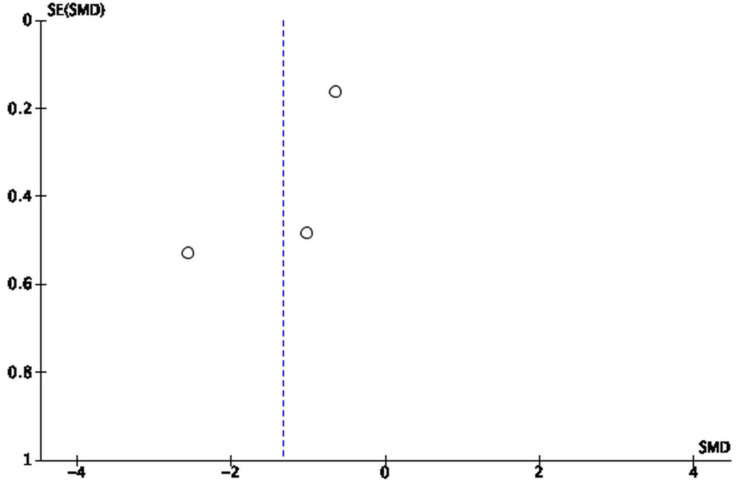
Forest plot for Tai Chi vs passive controls References: [23-26,30]

**Figure 16:**
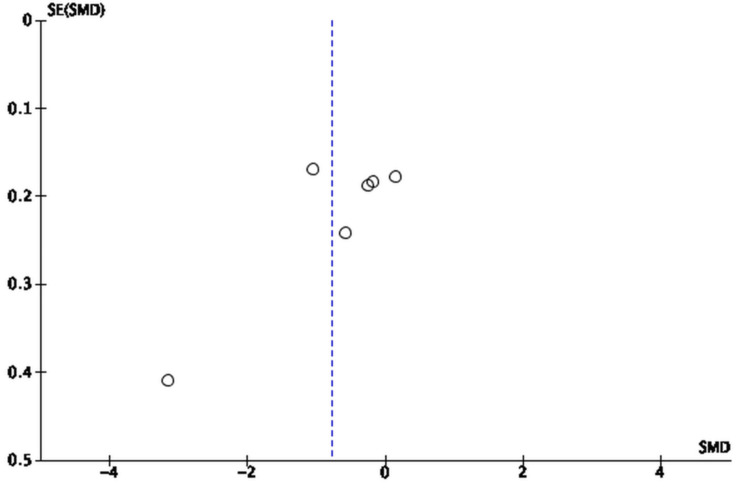
Funnel plot combined Qigong and Tai Chi exercises vs active and passive controls - Disability References: [24–26,30]

**Figure 17:**
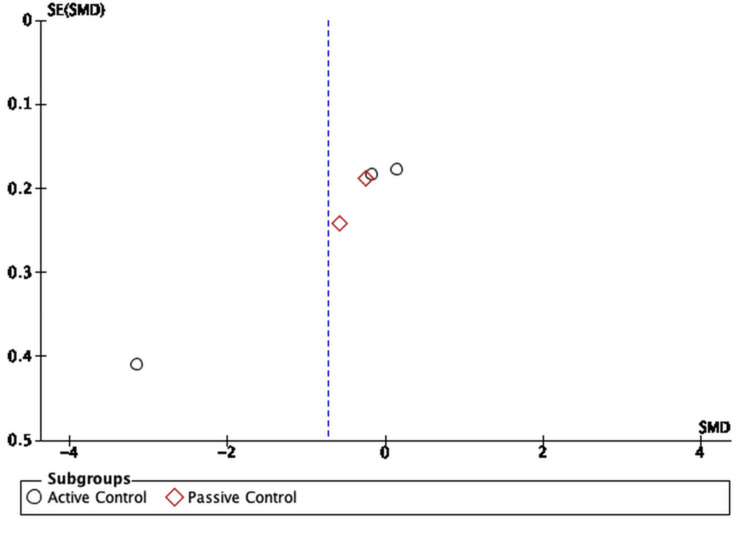
Funnel plot for Qigong vs active or passive controls – Disability

## Appendices

Appendix A: detailed search strategy

MEDLINE

(((((((Qigong) OR (Qi gong)) OR (Taichi)) OR (Tai Chi)) OR (Taiji)) OR (Tai Ji)) OR (Baduanjin)) AND (((((((low back pain) OR (lumbalgia)) OR (lumbago)) OR (backpain)) OR (dorsalgia)) OR (backache)) OR (low back ache))

Embase

"Qigong" OR "Qi gong" OR "Taichi" OR "Tai Chi"  OR "Taiji"  OR "Tai Ji"  OR "Baduanjin"

AND

"low back pain" OR "lumbalgia" OR "lumbago" OR "backpain" OR "dorsalgia" OR "backache" OR "low back ache"

PEDro

Therapy: no appropriate value in this field

Problem: Pain

Body part: Lumbar spine, sacro-iliac joint or pelvis

Subdiscipline:

Topic: Chronic pain

Method: Clinical Trial

When searching Match all search terms (AND)

Cochrane Library

(((((((Qigong) OR (Qi gong)) OR (Taichi)) OR (Tai Chi)) OR (Taiji)) OR (Tai Ji)) OR (Baduanjin)) AND (((((((low back pain) OR (lumbalgia)) OR (lumbago)) OR (backpain)) OR (dorsalgia)) OR (backache)) OR (low back ache))

Appendix B: risk of bias per domain and as percentage (intention to treat)

Appendix C: funnel plots
